# Obstetric Fistula Repair: Experience with Hospital-Based Outreach Approach in Nigeria

**DOI:** 10.5539/gjhs.v4n5p40

**Published:** 2012-07-05

**Authors:** Aniefiok J. Umoiyoho, Emmanuel C. Inyang-Etoh, Etiobong A. Etukumana

**Affiliations:** 1 Dept of obstetrics and gynecology, University of Uyo, Nigeria; 2 Dept of Family Medicine, University of Uyo, Nigeria

**Keywords:** obstetric fistula, patient selection, successful repair, experience, outreach approach

## Abstract

**Background:**

The huge back-log of obstetric fistula and the increasing incidence of the condition in Nigeria is a cause for concern for all stake-holders. This worrisome trend requires concerted effort with innovative strategies to redress the situation.

**Methods:**

Hospital-based outreach programs sponsored by a non-governmental organization with volunteer medical and health personnel were used to repair selected cases of obstetric fistula in Nigeria.

**Results:**

Fifty-two relatively simple obstetric fistulas were selected out of 68 (selection rate 76.5%) that presented for repair at 12 outreach programs in 5 different states of Nigeria. All the cases were repaired by one trained gynecological surgeon with a cure rate of 100%. The majority (50.0%) of the women were aged between 16 and 20 years with a mean age of 23.8 years ± 3.6. Most (80.9%) of the women in the study population were primiparous. The majority (50.0%) of the women were divorced at the time of their presentation for repair. A vast majority (76.9%) of the women had either primary level of education or no formal education. There was a preponderance (53.8%) of juxtacervical VVF among women in the study population.

**Conclusion:**

The use of hospital- based outreach approach to repair simple cases of obstetric fistula if multiplied could help reduce the large number of women living with unrepaired VVF in Nigeria.

## 1. Introduction

Obstetric fistula (OF), which often follows prolonged obstructed labor, is largely confined to developing countries of the world due to inadequate utilization of maternity services. The condition is characterized by total urinary incontinence, persistent perineal wetness, perineal excoriations and urinary stench (Karshinia, 2006; Danso, 2005). Women who are affected often lose their self-esteem and become socially withdrawn (Danso, 2005). Some of the women are neglected and abandoned by their spouses and close relations; in extreme cases, they are treated as outcast and ostracized from their communities (karshinia, 2006; Danso, 2005). Vesicovaginal fistula (VVF) poses considerable strain on the health of affected women because of the associated obnoxious features of the condition.

In 2001, 33,000 new cases of VVF were estimated to be added annually to the prevalent population in sub-Saharan Africa (UNFPA, 2006). Similarly, about 20,000 obstetric VVF cases were estimated to be added yearly to the already existing population of women living with VVF in Nigeria (UNFPA, 2006). Sequel to this rather worrisome trend, it would probably take several decades for the back-log of unrepaired cases of VVF to be cleared. This insinuation is buttressed by United Nation’s revelation that barely 33 surgeons provide fistula repair services in Nigeria and that only about 2500 cases of VVF are repaired annually in the country (Engender Health, 2003).

Vesicovaginal fistula may be simple and relatively easy to repair by trained surgeons or it could be otherwise complex with difficulties during repair. While simple cases could be repaired successfully by trained general or gynecological surgeons, complex ones require the skills and experience only available to expert fistula surgeons. Successful repair however does not require high medical technology but rather adequate clinical evaluation, correct diagnosis and patient selection. A health facility with a functional operating theatre and necessary surgical materials are however, indispensable (Arrowsmith, 1994; Gutman, 2007). Trained nurses and other ancillary health personnel are also required to ensure optimal postoperative care (Browning, 2004).

Many women who sustain VVF in Nigeria deliver or attempt to deliver in unlicensed maternity centers, where intrapartum care are often inadequate. These women shun hospital delivery because of illiteracy and ignorance about the benefits of delivering in a hospital; and the danger associated with delivery without a skilled attendant. Many of these women also cannot afford to pay the relatively high user fees charged in government approved maternity centres; therefore, they resort to unlicensed maternity homes and faith-based practitioners where less fees are charged (Etuk, 1999 & 2001). Such women when they develop VVF would also not afford to pay the service charge for VVF repair. They are therefore justifiably the beneficiaries of sponsored VVF repair through the outreach approach (Wall, 2011).

Considering the high prevalence and increasing incidence of obstetric fistula and the slow rate of repair in Nigeria, concerted effort is required with innovative strategies to increase the rate of repair. This study highlights our success with VVF repair through a hospital- based outreach approach, where a prognostic scoring system designed by the Authors was used in patient selection (Umoiyoho, 2011). We envisage that if this approach is multiplied in several other places, it could contribute significantly to reduce the number of women living with unrepaired obstetric fistula in Nigeria.

## 2. Subjects and Methods

### 2.1 Study Design and Setting

This was a prospective study that was designed to test the effectiveness of the proposed prognostic scoring system for the selection of patients for VVF repair based on available level of expertise. The study highlights our experience with VVF repair through hospital- based outreach programs, where all the patients repaired were first subjected to this selection criteria. The outreach programs were organized by Pro- Health International (PHI), a non-governmental health-oriented organization, which is funded by local and international donations from private individuals and organizations. The cardinal objective of PHI is to provide free but quality surgical care to people who cannot afford the cost of surgery in private and government-owned hospitals in developing countries.

Pro-Health International usually partners state governments which provide strategically located health facilities in their state for the outreach programs. While the government provides a functioning health facility with equipment and instruments, PHI mobilizes volunteer medical and health personnel as well as consumables and other materials needed for the smooth operation of the program. Members of the public in the catchment area were informed of the outreach through the electronic media and campaigns in churches, mosques, schools, markets, and villages, one month before the outreach. Areas of interest usually cover general surgery, gynaecological and ophthalmologic surgeries; but this also depends on the availability of volunteer-experts for these surgeries. Each outreach program usually last for about two weeks with the first two days used for the orientation of attending personnel, including resident medical and health personnel of the health facility used. Relevant resident personnel (doctors and nurses) were particularly instructed on the appropriate postoperative care following VVF repair.

This study covered a period of 3years during which time 12 outreach programs were carried out in five states of Nigeria. The states were Akwa Ibom, Cross River, Rivers and Adamawa states of Nigeria which had two outreach programs each. The fifth state, Taraba is located in north-eastern Nigeria and four outreach programs were carried out there because of the high prevalence of obstetric fistula.

### 2.2 Selection of Subjects

Fifty-two women who had relatively simple OF out of the 68 that presented were selected for repair based on their score with the prognostic scoring system that was designed by the Authors.(Umoiyoho, 2011) Sixteen women were therefore screened out because they had a score of 5 or more and their cases were adjudged to be complex thus requiring the services of an expert fistula surgeon. Patients who had scores of 4 or less were recruited into the study. The patients screened out included those who had the following characteristics detected during preliminary examination under anesthesia: size greater than 4cm, 3 previous attempts at repair, severe scarring and adherence of the fistula to the pubic bone. Others are those who had involvement of the urethra with circumferential defect, urethral loss and VVF combined with rectovaginal fistula. These patients were referred to the nearest regional fistula centre where the services of expert fistula surgeons were available.

The demographic characteristics of the patients were also obtained and recorded in their clinical folders. These characteristics included: age, parity, marital status, level of education and contact address. The location of the fistula in each patient was also noted. These pieces of information were later abstracted from the women’s clinical notes for the purpose of this study.

### 2.3 Data Analysis

Results were presented in the form of numerical, simple proportion and percentages. Some results were presented in tabular form for convenience. The data were analyzed using descriptive and inferential statistics.

### 2.4 Technique of Repair

All the repairs were performed under saddle block through a vaginal approach with the patient in the exaggerated Trendelenburg’s position. The introitus was exposed by anchoring the labia minora with stay stitches and the posterior vaginal wall retracted with the Sim’s vaginal speculum. The fistula was mobilized with the aid of long Alli’s tissue forceps and scar tissue excised. Relaxing incisions were made at the lateral vaginal walls to facilitate mobilization of the fistula where necessary. The fistula margin was dissected and separated from the vaginal wall and the edges freshened. The fistula was repaired without tension in two layers with polyglactin sutures (vicryl no.0). On completion of the repair, about 100ml of 1% methylene blue was instilled into the bladder to rule out any leakage at the repair site. A satisfactory outcome was followed by closure of the vaginal mucosa in a perpendicular plane to the suture line of the fistula repair. A three-way Foley catheter, size 18F was passed and strapped to the thigh for continuous bladder drainage. This rather large caliber of urethral catheter was used to facilitate continuous drainage and prevent blockage of the eye of the catheter with blood clots or bladder debris. De-Pezzer catheter, which would have been ideal was not available. Each patient had 4 litres of intravenous fluid every 24 hours for the first 72 hours and thereafter liberal oral fluid intake. Urinary output was monitored to ensure that this was adequate. Attention was also paid to the functioning of the catheter-urine bag system to prevent any interruption of continuous bladder drainage. Every patient also received antibiotics and analgesics as needed postoperatively. Vulvovaginal toileting was done where necessary and early ambulation was encouraged for all the patients. The urethral catheter was retained for 14 days after which time, bladder training was commenced and the catheter finally removed following successful bladder training. The postoperative treatment was clearly outlined in the case notes of the patients to guide the resident medical officers of the hospital on further postoperative management of each patient. Patients were discharged following successful bladder training and seen postoperatively 6 weeks following the repair and again at 12 weeks. During this time, they were strongly advised to abstain from sexual intercourse. Cases of failure were to be referred to the nearest regional fistula centre for further evaluation and repair; although, this was not necessary since none of the cases repaired in this series failed. All the patients who remained continent of urine by the 6week follow up visit were certified cured and on the 12 week follow up visit, they were placed on a method of contraception and discharged from follow up. The senior Author visited all the centres 12 weeks after repair to ascertain the final outcome for each patient that was repaired. All the women were counseled on the need to postpone pregnancy at least for a period of one year following the repair. The need for all subsequent pregnancies to be delivered by elective caesarean section was also stressed.

## 3. Results

Sixty-eight women with VVF presented at 12 of the outreach programs for repair at an average of 4 women per outreach program. Fifty-two (73.5%) of them who scored 4 or less using the proposed prognostic scoring system were assessed to be relatively simple, and so, selected for repair based on available level of surgical expertise. This gave a selection rate of 76.5%. The repair was done by the senior Author, a gynecological surgeon, who had been duly trained on VVF repair. The cure rate of repair for these selected cases was 100%. The same surgical technique was used for all the cases.

The table shows the age-groups of women in the study population. The majority (50.0%) of the women were aged between 16 and 20 years. The mean age of women in the study population was 23.8 years ± 3.5.

The parity of women in the study population showed that most (80.9%) of the women were primiparous.

The marital status of women in the study population revealed that the majority (50.0%) of the women were divorced by their husbands as against 38.5% of the women who were still married at the time of the study.

The level of education of the women shows that a vast majority (76.9%) of the women had either primary level of education or no formal education. None of the women in the study population attained tertiary level of education.

The location of the fistula in the genital tract among women in the study population shows the majority (53.8%) of the fistulas being juxtacervical.

**Table:** 

VARIABLES	No. (%)
Age groups	
16-20	26 (50.0)
21-25	10 (19.2)
26-30	6 (11.6)
31-35	2 (3.8)
36-40	2 (3.8)
41-45	6 (11.6)
Parity	
P1	42 (80.9)
P2	2 (3.8)
P3	2 (3.8)
P4	2 (3.8)
>P5	4 (7.7)
Marital status	
Single	6 (11.5)
Married	20 (38.5)
Divorced	26 (50.0)
Level of education	
No formal education	8 (15.4)
Primary	32 (61.5)
Secondary	12 (23.1)
Tertiary	0 (0.0)
Location of VVF	
Juxtaurethral	12 (23.1)
Midvaginal	12 (23.1)
Juxtacervical	28 (53.8)
Total no. of women	52 (100.0%)

## 4. Discussion

The need for all stakeholders to close ranks and explore innovative ways to reduce the huge backlog of unrepaired obstetric fistula in Nigeria has become imperative. The hospital- based outreach approach has proved promising in this regard, provided patient selection is done and the VVF is categorized based on the level of expertise of the attending surgeons. This approach is in compliance with the recommendation of the World Health Organization that VVF be classified into simple and complex types based on available expertise (De Bernis, 2007). The cure rate of 100% following repair of the VVF in this series was exceptional and a testimony to the effectiveness of the prognostic scoring system proposed by Umoiyoho (2011). A comparable cure rate of 97.7% was obtained by Ward^11^ et al in their series apparently because the Authors were expert fistula surgeons. Lower cure rates of 88%, 87.1% and 75% are usual and were obtained in two other Nigerian studies and Norway respectively, where patient selection was not considered and the authors were not expert fistula surgeons (Kelly, 1993; Kullima, 2009). The need for patient selection is also supported by the fact that the number of previous attempts at repair influences negatively the prospect of success (Arrowsmith, 1994; Umoiyoho, 2011).

The modal age-group in this study was 16 to 20years. This finding has once again confirmed the association between obstetric fistula and obstructed labor, which are prevalent in teenage mothers because of incompletely developed pelvis and the resulting cephalopelvic disproportion (Danso, 2005). A vast majority of women with VVF in Zaria, Nigeria were also teenagers in consonance with our finding (Tahzib, 1983). This study also revealed a mean age of 23.8years, very similar to the mean age of 24years obtained in Benin, Nigeria (Gharoro, 2009). Results from Niger republic and Benin- city, Nigeria however contrasted with our findings; with the mean age of 27.3years and 31years respectively (Ndiaye, 2009). The deduction from these results is however the realization that obstetric fistula afflict largely young women in the prime of their reproductive life.

The modal parity (80.9%) among women in the study population was para 1. This result is a reflection of the preponderance of teenage mothers in the study population and other young women who are merely starting their reproductive career. Studies done in northern Nigeria also found primiparous women comprising the majority of the women in their series, 51.3% and 52.0% respectively (Kullima, 2009; Tahzib, 1983). In contrast, a study done in Uyo, Nigeria found only 31.4% primiparous women in their series (Hilton, 1998).

This study has revealed that 26% of the women were divorced by their husbands at the time of presentation for repair of their fistula. This finding is not unusual as it supports the fact that most afflicted women suffer neglect and abandonment by close relations including their husbands (Karshinia, 2006; Danso, 2005). A comparable divorce rate of 25% was obtained by Gharoro and Agholor (2009). Kullima et al 2009, in contrast, found a much higher divorce rate of 50% in their series in north-eastern Nigeria.

The majority (76.9%) of the women were either educated to the primary level or had no formal education. The level of education of women has been found to influence positively their attitude towards utilization of health facilities (Olusanya, 1985). A woman with a higher level of education would likely avoid VVF by electing to deliver in a hospital. This finding has also confirmed the results obtained from other Nigerian studies which showed that the vast majority of women who sustain VVF have low level of education (Hilton, 1998; Kullima, 2009; Tahzib, 1983).

There was a preponderance (53.8%) of juxtacervical VVF in the study population. The reason for this rather unusual finding has not been adduced by this study, although, it contrasted with the results of a review of VVF in Ibadan and Zaria, where 60% of the VVF were midvaginal fistula and only 15% were juxtacervical (Ojengbede, 2007).

In conclusion, the prognostic scoring system designed by Umoiyoho and Inyang-Etoh is effective in the selection of simple cases of VVF for repair through hospital- based outreach programs by trained fistula surgeons in regions with a high prevalence of unrepaired vesicovaginal fistula. A multiplication of this approach could go a long way to reduce the hitherto huge back-log of unrepaired obstetric fistula in Nigeria.
